# Supplementary material for: Synergistic Antimicrobial Activity of Silver Nanoparticles with an Emergent Class of Azoimidazoles
Source: Pharmaceutics. 2023 Mar 13;15(3):926. doi: 10.3390/pharmaceutics15030926 (PMC10053004; doi:10.3390/pharmaceutics15030926)
Supplement: Supplementary file 1 [file pharmaceutics-15-00926-s001.zip › pharmaceutics-2238947-supplementary.pdf]

pharmaceutics

Supporting Information

Synergistic Antimicrobial Activity of Silver Nanoparticles with an Emergent Class of Azoimidazoles

Ana Isabel Ribeiro [1], Bárbara Vieira [1], Daniela Dantas [2], Bárbara Silva [3,4,5], Eugénia Pinto [3,6,*], Fátima Cerqueira [7,8,9], Renata Silva [4,5], Fernando Remião [4,5], Jorge Padrão [1], Alice Maria Dias [2] and Andrea Zille [1,*]

1 Centre for Textile Science and Technology (2C2T), Department of Textile Engineering, University of Minho, Campus of Azurém, 4800-058 Guimarães, Portugal
2 Department of Chemistry, Chemistry Centre of University of Minho (CQUM), University of Minho, Campus of Gualtar, 4710-057 Braga, Portugal
3 Laboratory of Microbiology, Biological Sciences Department, Faculty of Pharmacy, University of Porto, 4050-313 Porto, Portugal
4 UCIBIO—Applied Molecular Biosciences Unit, REQUIMTE, Laboratory of Toxicology, Department of Biological Sciences, Faculty of Pharmacy, University of Porto, Rua de Jorge Viterbo Ferreira n° 228, 4050-313 Porto, Portugal
5 Associate Laboratory i4HB—Institute for Health and Bioeconomy, Faculty of Pharmacy, University of Porto, 4050-313 Porto, Portugal
6 CIIMAR/CIMAR, Interdisciplinary Centre of Marine and Environmental Research, Terminal de Cruzeiros do Porto de Leixões, 4450-208 Matosinhos, Portugal
7 Molecular Oncology and Viral Pathology Group, Research Center of IPO Porto (CI-IPOP)/RISE@CI-IPOP (Health Research Network), Portuguese Oncology Institute of Porto (IPO Porto)/Porto Comprehensive Cancer Center (Porto.CCC), 4200-072 Porto, Portugal
8 Faculty of Health Sciences, Fernando Pessoa University, 4200-150 Porto, Portugal
9 Instituto de Investigação, Inovação e Desenvolvimento (FP-I3ID), Biomedical and Health Sciences Research Unit (FP-BHS), Universidade Fernando Pessoa, Praça 9 de Abril, 349, 4249-004 Porto, Portugal
* Correspondence: epinto@ff.up.pt (E.P.); azille@2c2t.uminho.pt (A.Z.)

SI-Table S1. Checkerboard results by combining Amz and AgNPs. Synergism – green; Additive – yellow; Indiferent or antagonist – white.

		S. aureus				*E. coli*				*C. albicans*				*C. krusei*			
[µg·mL^{-1}]		AgNPs.Cit		AgNPs.PVP		AgNPs.Cit		AgNPs.PVP		AgNPs.Cit		AgNPs.PVP		AgNPs.Cit		AgNPs.PVP	
Amz	NPs	Growth (%)	FICI	Growth (%)	FICI	Growth (%)	FICI	Growth (%)	FICI	Growth (%)	FICI	Growth (%)	FICI	Growth (%)	FICI	Growth (%)	FICI
128.0	1.6	1.2	1.0	3.2	1.0	1.9	1.0	4.2	1.0	0	16.1	2.7	16.1	8.9	8.2	7.0	8.1
128.0	3.1	0	1.0	0	1.0	0	1.0	0	1.0	4.3	16.3	7.7	16.3	16.6	8.5	9.8	8.1
128.0	6.3	0.4	1.1	8.2	1.0	3.9	1.1	6.7	1.0	3.4	16.5	0	16.5	12.3	9.0	9.0	8.3
128.0	12.5	0.7	1.1	0	1.0	9.0	1.1	0	1.0	8.7	17.0	0	17.0	12.6	10.0	7.5	8.5
128.0	25	2.1	1.3	8.6	1.0	4.6	1.3	6.0	1.0	16.4	18.0	4.5	18.0	35.8	12.0	19.3	9.0
64.0	1.6	4.7	0.5	4.4	0.5	11.5	0.5	8.7	0.5	0	8.1	2.4	8.1	3.7	4.2	4.9	4.1
64.0	3.1	4.2	0.5	0	0.5	1.9	0.5	2.3	0.5	0	8.3	3.0	8.3	0	4.5	8.5	4.1
64.0	6.3	5.1	0.6	11.0	0.5	1.4	0.6	1.5	0.5	0	8.5	1.8	8.5	1.1	5.0	6.2	4.3
64.0	12.5	4.9	0.6	7.8	0.5	2.6	0.6	0.3	0.5	0	9.0	8.0	9.0	7.2	6.0	8.8	4.5
64.0	25	3.1	0.8	0	0.5	0	0.8	0	0.5	7.1	10.0	0	10.0	5.7	8.0	7.5	5.0
32.0	1.6	15.6	0.3	38.1	0.3	0.3	0.3	50.6	0.3	0	4.1	2.1	4.1	10.6	2.2	6.4	2.1
32.0	3.1	12.6	0.3	33.7	0.3	0.5	0.3	42.5	0.3	0	4.3	1.8	4.3	8.3	2.5	7.7	2.1
32.0	6.3	11.2	0.3	38.7	0.3	50.5	0.3	52.9	0.3	0	4.5	4.2	4.5	9.7	3.0	7.7	2.3
32.0	12.5	11.0	0.4	24.8	0.3	1.5	0.4	53.6	0.3	0	5.0	0	5.0	12.0	4.0	6.2	2.5
32.0	25	10.7	0.5	9.8	0.3	1.7	0.5	47.5	0.3	13.0	6.0	3.3	6.0	19.5	6.0	8.8	3.0
16.0	1.6	20.0	0.1	65.5	0.1	0.6	0.1	70.3	0.1	0	2.1	1.2	2.1	8.6	1.2	9.5	1.1
16.0	3.1	17.4	0.2	54.9	0.1	3.9	0.2	1.6	0.1	1.2	2.3	3.6	2.3	10.0	1.5	9.8	1.1
16.0	6.3	14.5	0.2	57.9	0.1	69.7	0.2	69.3	0.1	3.7	2.5	3.9	2.5	9.5	2.0	10.8	1.3
16.0	12.5	13.5	0.3	42.7	0.1	68.1	0.3	63.2	0.1	6.8	3.0	3.9	3.0	12.9	3.0	8.5	1.5
16.0	25	9.9	0.4	25.1	0.1	54.8	0.4	54.9	0.1	24.5	4.0	0.3	4.0	21.8	5.0	13.1	2.0
8.0	1.6	25.9	0.1	79.8	0.1	79.2	0.1	80.9	0.1	3.1	1.1	4.2	1.1	15.2	0.7	14.4	0.6
8.0	3.1	22.7	0.1	72.5	0.1	76.3	0.1	74.3	0.1	3.7	1.3	4.2	1.3	13.8	1.0	13.9	0.6
8.0	6.3	21.1	0.1	69.1	0.1	79.0	0.1	10.4	0.1	2.2	1.5	3.6	1.5	14.0	1.5	12.6	0.8
8.0	12.5	19.6	0.2	64.9	0.1	74.1	0.2	2.7	0.1	0	2.0	5.9	2.0	16.6	2.5	12.1	1.0
8.0	25	13.1	0.3	38.3	0.1	2.6	0.3	66.8	0.1	0	3.0	1.2	3.0	21.5	4.5	17.0	1.5
4.0	1.6	28.8	0.0	86.0	0.0	90.6	0.0	88.7	0.0	5.0	0.6	8.0	0.6	38.7	0.5	23.7	0.3
4.0	3.1	24.4	0.1	85.0	0.0	3.1	0.1	86.1	0.0	5.9	0.8	7.1	0.8	38.7	0.8	28.1	0.4
4.0	6.3	23.7	0.1	81.2	0.0	83.7	0.1	83.7	0.0	6.2	1.0	6.8	1.0	39.0	1.3	25.5	0.5
4.0	12.5	21.5	0.2	69.9	0.0	77.7	0.2	1.5	0.0	0.3	1.5	5.6	1.5	38.4	2.3	27.3	0.8
4.0	25	14.9	0.3	40.1	0.0	59.9	0.3	18.8	0.0	2.2	2.5	5.9	2.5	40.4	4.3	28.6	1.3
2.0	1.6	30.6	0.0	96.4	0.0	94.6	0.0	100.6	0.0	9.3	0.4	13.1	0.4	97.1	0.4	87.6	0.2
2.0	3.1	28.1	0.0	98.0	0.0	91.2	0.0	88.7	0.0	9.3	0.5	12.2	0.5	90.8	0.6	89.4	0.3
2.0	6.3	26.6	0.1	91.0	0.0	83.6	0.1	88.6	0.0	5.6	0.8	11.9	0.8	82.8	1.1	81.2	0.4
2.0	12.5	22.2	0.1	71.9	0.0	77.3	0.1	78.1	0.0	8.0	1.3	6.2	1.3	34.7	2.1	75.3	0.6
2.0	25	15.2	0.3	52.1	0.0	0	0.3	73.8	0.0	1.2	2.3	7.1	2.3	15.8	4.1	57.0	1.1

SI-Table S2. Checkerboard results by combining Azolz and AgNPs. Synergism – green; Additive – yellow; Indiferent or antagonist – white.

		S. aureus				*E. coli*				*C. albicans*				*C. krusei*			
[μg·mL^{-1}]		AgNPs.Cit		AgNPs.PVP		AgNPs.Cit		AgNPs.PVP		AgNPs.Cit		AgNPs.PVP		AgNPs.Cit		AgNPs.PVP	
Azolz	NPs	Growth (%)	FICI	Growth (%)	FICI	Growth (%)	FICI	Growth (%)	FICI	Growth (%)	FICI	Growth (%)	FICI	Growth (%)	FICI	Growth (%)	FICI
128.0	1.6	2.2	1.0	4.7	1.0	1.0	1.0	0.9	1.0	3.8	32.1	8.0	32.1	6.9	16.2	8.0	16.1
128.0	3.1	0	1.0	3.3	1.0	2.3	1.0	5.6	1.0	16.5	32.3	9.0	32.3	15.2	16.5	16.2	16.1
128.0	6.3	12.5	1.1	2.1	1.0	8.6	1.1	0	1.0	2.4	32.5	9.4	32.5	11.2	17.0	7.4	16.3
128.0	12.5	4.8	1.1	3.8	1.0	7.4	1.1	7.8	1.0	30.7	33.0	17.5	33.0	27.1	18.0	33.0	16.5
128.0	25	29.7	1.3	2.8	1.0	1.2	1.3	5.1	1.0	0	34.0	0.9	34.0	25.3	20.0	6.6	17.0
64.0	1.6	25.7	0.5	26.4	0.5	2.4	0.5	43.4	0.5	11.8	16.1	8.0	16.1	15.4	8.2	8.5	8.1
64.0	3.1	24.4	0.5	23.8	0.5	1.2	0.5	5.6	0.5	9.9	16.3	28.8	16.3	14.6	8.5	20.1	8.1
64.0	6.3	17.0	0.6	26.6	0.5	25.5	0.6	4.9	0.5	3.8	16.5	7.5	16.5	14.6	9.0	9.1	8.3
64.0	12.5	12.9	0.6	25.7	0.5	0	0.6	3.3	0.5	21.2	17.0	7.5	17.0	5.9	10.0	4.9	8.5
64.0	25	0	0.8	23.8	0.5	0	0.8	12.5	0.5	15.6	18.0	16.5	18.0	26.3	12.0	7.4	9.0
32.0	1.6	42.7	0.3	56.9	0.3	3.4	0.3	78.6	0.3	9.0	8.1	9.9	8.1	12.8	4.2	10.2	4.1
32.0	3.1	41.9	0.3	53.6	0.3	1.4	0.3	75.6	0.3	5.7	8.3	3.8	8.3	7.7	4.5	9.1	4.1
32.0	6.3	40.1	0.3	55.7	0.3	48.3	0.3	47.6	0.3	12.3	8.5	12.3	8.5	13.6	5.0	9.1	4.3
32.0	12.5	32.5	0.4	46.7	0.3	50.8	0.4	46.7	0.3	9.0	9.0	21.7	9.0	8.8	6.0	11.3	4.5
32.0	25	34.9	0.5	44.8	0.3	30.9	0.5	25.7	0.3	36.3	10.0	6.1	10.0	40.4	8.0	10.2	5.0
16.0	1.6	64.5	0.1	74.8	0.1	59.6	0.1	84.1	0.1	9.0	4.1	8.5	4.1	13.3	2.2	10.4	2.1
16.0	3.1	62.1	0.2	73.4	0.1	46.9	0.2	85.6	0.1	3.3	4.3	6.6	4.3	11.2	2.5	10.7	2.1
16.0	6.3	59.9	0.2	75.0	0.1	3.6	0.2	59.0	0.1	3.3	4.5	5.7	4.5	8.0	3.0	13.2	2.3
16.0	12.5	59.0	0.3	66.4	0.1	63.0	0.3	51.8	0.1	0	5.0	7.5	5.0	0	4.0	9.9	2.5
16.0	25	32.7	0.4	52.4	0.1	35.8	0.4	40.1	0.1	0	6.0	3.3	6.0	2.7	6.0	13.5	3.0
8.0	1.6	72.8	0.1	87.2	0.1	88.2	0.1	100.2	0.1	7.5	2.1	5.2	2.1	14.1	1.2	15.4	1.1
8.0	3.1	70.2	0.1	86.7	0.1	71.1	0.1	94.1	0.1	8.5	2.3	7.5	2.3	12.8	1.5	13.7	1.1
8.0	6.3	69.0	0.1	85.7	0.1	64.7	0.1	88.4	0.1	9.9	2.5	7.1	2.5	17.0	2.0	14.3	1.3
8.0	12.5	59.8	0.2	75.2	0.1	41.5	0.2	72.2	0.1	14.2	3.0	6.6	3.0	17.3	3.0	12.4	1.5
8.0	25	43.0	0.3	59.8	0.1	55.3	0.3	48.3	0.1	15.1	4.0	11.3	4.0	42.6	5.0	19.2	2.0
4.0	1.6	75.9	0.0	91.7	0.0	100.6	0.0	71.3	0.0	9.0	1.1	8.5	1.1	20.2	0.7	18.4	0.6
4.0	3.1	72.5	0.1	93.4	0.0	98.8	0.1	84.5	0.0	12.7	1.3	9.0	1.3	17.6	1.0	21.7	0.6
4.0	6.3	73.1	0.1	99.5	0.0	72.4	0.1	84.9	0.0	13.2	1.5	7.1	1.5	14.4	1.5	21.2	0.8
4.0	12.5	65.8	0.2	84.5	0.0	8.5	0.2	77.4	0.0	25.0	2.0	9.4	2.0	19.7	2.5	19.5	1.0
4.0	25	67.3	0.3	69.1	0.0	49.8	0.3	55.6	0.0	41.0	3.0	18.4	3.0	33.2	4.5	20.1	1.5
2.0	1.6	80.6	0.0	100.5	0.0	103.0	0.0	70.1	0.0	16.5	0.6	17.0	0.6	75.0	0.5	89.6	0.3
2.0	3.1	77.8	0.0	102.4	0.0	102.1	0.0	67.6	0.0	14.6	0.8	12.3	0.8	26.9	0.8	87.1	0.4
2.0	6.3	73.9	0.1	96.9	0.0	83.5	0.1	90.9	0.0	17.5	1.0	14.6	1.0	13.0	1.3	80.8	0.5
2.0	12.5	67.5	0.1	89.5	0.0	79.8	0.1	80.6	0.0	29.2	1.5	10.8	1.5	11.2	2.3	77.5	0.8
2.0	25	62.6	0.3	70.3	0.0	9.3	0.3	48.6	0.0	21.7	2.5	12.7	2.5	11.2	4.3	19.5	1.3

SI-Table S3. Checkerboard results by combining AzoIz.DMA and AgNPs. Synergism – green; Additive – yellow; Indiferent or antagonist – white.

		S. aureus				*E. coli*				C. albicans				C. krusei			
[μg·mL^{-1}]		AgNPs.Cit		AgNPs.PVP		AgNPs.Cit		AgNPs.PVP		AgNPs.Cit		AgNPs.PVP		AgNPs.Cit		AgNPs.PVP	
AzoIz. DMA	NPs	Growth (%)	FICI	Growth (%)	FICI	Growth (%)	FICI	Growth (%)	FICI	Growth (%)	FICI	Growth (%)	FICI	Growth (%)	FICI	Growth (%)	FICI
128.0	1.6	0	2.0	4.2	2.0	6.8	1.0	0	1.0	0	16.1	10.4	16.1	0.3	16.2	16.4	16.1
128.0	3.1	0	2.0	15.1	2.0	0	1.0	0	1.0	5.9	16.3	0	16.3	14.2	16.5	0	16.1
128.0	6.3	0	2.1	5.6	2.0	0	1.1	0	1.0	6.7	16.5	1.0	16.5	10.2	17.0	6.8	16.3
128.0	12.5	5.4	2.1	21.0	2.0	0	1.1	0	1.0	9.5	17.0	16.0	17.0	0	18.0	8.6	16.5
128.0	25	0	2.3	8.6	2.0	0	1.3	2.0	1.0	0	18.0	18.6	18.0	16.5	20.0	28.1	17.0
64.0	1.6	0	1.0	0	1.0	0	0.5	19.4	0.5	0	8.1	0	8.1	7.3	8.2	0.5	8.1
64.0	3.1	0	1.0	0	1.0	0	0.5	15.2	0.5	0	8.3	1.5	8.3	6.3	8.5	7.8	8.1
64.0	6.3	0	1.1	0	1.0	0	0.6	0	0.5	0	8.5	0	8.5	1.8	9.0	2.9	8.3
64.0	12.5	0	1.1	0	1.0	0	0.6	0	0.5	2.5	9.0	2.9	9.0	7.1	10.0	5.5	8.5
64.0	25	0	1.3	0	1.0	0	0.8	0	0.5	16.8	10.0	1.0	10.0	39.9	12.0	10.9	9.0
32.0	1.6	27.1	0.5	8.9	0.5	64.5	0.3	70.7	0.3	0	4.1	0	4.1	7.6	4.2	6.5	4.1
32.0	3.1	14.7	0.5	13.3	0.5	64.8	0.3	71.2	0.3	0	4.3	0.5	4.3	8.7	4.5	8.8	4.1
32.0	6.3	19.4	0.6	12.7	0.5	56.5	0.3	57.1	0.3	0	4.5	0	4.5	10.8	5.0	5.2	4.3
32.0	12.5	29.0	0.6	0	0.5	60.0	0.4	44.9	0.3	0	5.0	2.7	5.0	9.4	6.0	9.6	4.5
32.0	25	0	0.8	0	0.5	5.5	0.5	23.8	0.3	30.0	6.0	3.1	6.0	35.4	8.0	10.9	5.0
16.0	1.6	65.6	0.3	50.2	0.3	90.6	0.1	82.2	0.1	0.8	2.1	0.0	2.1	11.0	2.2	9.6	2.1
16.0	3.1	53.8	0.3	45.4	0.3	93.5	0.2	88.5	0.1	0	2.3	0.5	2.3	9.4	2.5	9.4	2.1
16.0	6.3	48.3	0.3	44.5	0.3	90.6	0.2	84.0	0.1	0	2.5	1.7	2.5	7.3	3.0	10.4	2.3
16.0	12.5	60.2	0.4	38.3	0.3	91.8	0.3	78.2	0.1	0	3.0	1.2	3.0	9.2	4.0	10.1	2.5
16.0	25	12.0	0.5	19.1	0.3	42.6	0.4	47.6	0.1	28.0	4.0	5.3	4.0	37.0	6.0	13.5	3.0
8.0	1.6	96.9	0.1	67.8	0.1	98.8	0.1	90.8	0.1	2.8	1.1	4.4	1.1	16.0	1.2	13.0	1.1
8.0	3.1	79.8	0.2	66.7	0.1	97.5	0.1	92.3	0.1	1.1	1.3	4.1	1.3	12.1	1.5	13.8	1.1
8.0	6.3	72.3	0.2	63.4	0.1	94.9	0.1	85.0	0.1	1.1	1.5	3.1	1.5	8.9	2.0	12.5	1.3
8.0	12.5	72.6	0.3	52.8	0.1	98.9	0.2	76.9	0.1	0.0	2.0	3.1	2.0	8.1	3.0	11.9	1.5
8.0	25	44.3	0.4	33.9	0.1	68.9	0.3	50.9	0.1	1.7	3.0	3.6	3.0	38.8	5.0	15.1	2.0
4.0	1.6	115.5	0.1	75.6	0.1	101.1	0.0	83.7	0.0	5.6	0.6	7.7	0.6	51.7	0.7	48.1	0.6
4.0	3.1	100.0	0.1	78.8	0.1	105.7	0.1	92.7	0.0	3.9	0.8	8.0	0.8	15.5	1.0	52.5	0.6
4.0	6.3	85.3	0.1	73.5	0.1	96.4	0.1	85.7	0.0	2.5	1.0	8.0	1.0	11.8	1.5	48.3	0.8
4.0	12.5	83.4	0.2	63.2	0.1	99.0	0.2	84.2	0.0	3.1	1.5	6.3	1.5	15.7	2.5	31.9	1.0
4.0	25	33.2	0.3	45.4	0.1	64.7	0.3	45.3	0.0	0	2.5	8.5	2.5	29.1	4.5	10.6	1.5
2.0	1.6	125.5	0.0	84.6	0.0	101.5	0.0	65.9	0.0	11.8	0.4	18.4	0.4	81.9	0.5	89.9	0.3
2.0	3.1	114.1	0.1	86.7	0.0	106.3	0.0	84.5	0.0	9.2	0.5	16.2	0.5	26.2	0.8	87.3	0.4
2.0	6.3	105.4	0.1	79.9	0.0	100.7	0.1	71.5	0.0	10.1	0.8	10.9	0.8	9.7	1.3	76.4	0.5
2.0	12.5	85.3	0.2	69.3	0.0	95.7	0.1	79.0	0.0	9.2	1.3	7.7	1.3	10.5	2.3	64.2	0.8
2.0	25	40.1	0.3	54.3	0.0	56.3	0.3	47.9	0.0	7.6	2.3	9.0	2.3	22.0	4.3	13.5	1.3

SI-Table S4. Checkerboard results by combining AzoIz.Pip and AgNPs. Synergism – green; Additive – yellow; Indiferent or antagonist – white.

		S. aureus				*E. coli*				C. albicans				C. krusei			
[µg·mL^{-1}]		AgNPs.Cit		AgNPs.PVP		AgNPs.Cit		AgNPs.PVP		AgNPs.Cit		AgNPs.PVP		AgNPs.Cit		AgNPs.PVP	
AzoIz. Pip	NPs	Growth (%)	FICI	Growth (%)	FICI	Growth (%)	FICI	Growth (%)	FICI	Growth (%)	FICI	Growth (%)	FICI	Growth (%)	FICI	Growth (%)	FICI
128.0	1.6	8.5	2.0	0	2.0	27.5	2.0	36.0	2.0	42.9	32.1	45.2	32.1	29.8	16.2	27.2	16.1
128.0	3.1	41.6	2.0	0	2.0	32.3	2.0	16.2	2.0	1.2	32.3	77.4	32.3	24.7	16.5	22.6	16.1
128.0	6.3	41.8	2.1	28.0	2.0	22.8	2.1	11.8	2.0	0	32.5	70.3	32.5	19.9	17.0	18.2	16.3
128.0	12.5	7.5	2.1	39.4	2.0	42.1	2.1	20.4	2.0	61.3	33.0	125.2	33.0	17.7	18.0	16.2	16.5
128.0	25	33.5	2.3	13.4	2.0	27.6	2.3	12.4	2.0	1.8	34.0	175.5	34.0	66.0	20.0	60.3	17.0
64.0	1.6	0	1.0	0	1.0	0	1.0	0	1.0	25.6	16.1	5.8	16.1	0	8.2	0	8.1
64.0	3.1	0	1.0	0	1.0	0	1.0	0	1.0	8.3	16.3	0	16.3	0.3	8.5	0.3	8.1
64.0	6.3	0	1.1	0	1.0	0	1.1	0	1.0	0	16.5	23.2	16.5	-6.2	9.0	0	8.3
64.0	12.5	0	1.1	0	1.0	0	1.1	0	1.0	38.7	17.0	38.1	17.0	12.9	10.0	11.8	8.5
64.0	25	0	1.3	0	1.0	0	1.3	0	1.0	45.8	18.0	16.8	18.0	10.1	12.0	9.2	9.0
32.0	1.6	0.4	0.5	5.0	0.5	42.3	0.5	54.6	0.5	22.6	8.1	0	8.1	0	4.2	0	4.1
32.0	3.1	2.2	0.5	1.6	0.5	40.9	0.5	50.7	0.5	20.2	8.3	0	8.3	0	4.5	0	4.1
32.0	6.3	0	0.6	0	0.5	34.0	0.6	42.5	0.5	0.6	8.5	0	8.5	0	5.0	0	4.3
32.0	12.5	3.2	0.6	0	0.5	32.1	0.6	40.7	0.5	0	9.0	0	9.0	0	6.0	0	4.5
32.0	25	0	0.8	0	0.5	0	0.8	14.6	0.5	0	10.0	0	10.0	0.3	8.0	0.3	5.0
16.0	1.6	51.9	0.3	50.0	0.3	72.2	0.3	84.8	0.3	10.7	4.1	0	4.1	0	2.2	0	2.1
16.0	3.1	49.1	0.3	49.4	0.3	66.5	0.3	87.6	0.3	7.1	4.3	0	4.3	0	2.5	0	2.1
16.0	6.3	47.5	0.3	53.4	0.3	61.1	0.3	77.3	0.3	0	4.5	0	4.5	0	3.0	0.5	2.3
16.0	12.5	49.5	0.4	41.4	0.3	61.4	0.4	68.7	0.3	0	5.0	0	5.0	0	4.0	0	2.5
16.0	25	10.3	0.5	18.6	0.3	20.5	0.5	39.4	0.3	0	6.0	11.0	6.0	5.1	6.0	4.6	3.0
8.0	1.6	76.4	0.1	72.2	0.1	84.3	0.1	96.0	0.1	6.5	2.1	0	2.1	5.6	1.2	5.1	1.1
8.0	3.1	71.7	0.2	76.0	0.1	79.1	0.2	92.7	0.1	11.3	2.3	0	2.3	4.5	1.5	4.1	1.1
8.0	6.3	79.6	0.2	72.4	0.1	75.7	0.2	89.1	0.1	11.9	2.5	0	2.5	5.1	2.0	4.6	1.3
8.0	12.5	75.4	0.3	75.0	0.1	77.4	0.3	79.5	0.1	8.9	3.0	0	3.0	2.8	3.0	2.6	1.5
8.0	25	57.0	0.4	41.6	0.1	44.4	0.4	58.3	0.1	36.9	4.0	0	4.0	7.0	5.0	6.4	2.0
4.0	1.6	87.1	0.1	85.8	0.1	93.0	0.1	102.2	0.1	7.1	1.1	4.5	1.1	16.0	0.7	14.6	0.6
4.0	3.1	79.8	0.1	79.6	0.1	83.5	0.1	102.8	0.1	8.3	1.3	8.4	1.3	11.2	1.0	10.3	0.6
4.0	6.3	84.6	0.1	81.6	0.1	79.7	0.1	99.9	0.1	8.3	1.5	0	1.5	11.0	1.5	10.0	0.8
4.0	12.5	79.4	0.2	77.0	0.1	75.3	0.2	88.7	0.1	3.6	2.0	1.3	2.0	10.7	2.5	9.7	1.0
4.0	25	66.1	0.3	57.0	0.1	45.7	0.3	60.5	0.1	25.6	3.0	0	3.0	13.8	4.5	12.6	1.5
2.0	1.6	93.7	0.0	86.4	0.0	95.4	0.0	94.7	0.0	6.5	0.6	11.0	0.6	91.9	0.5	83.8	0.3
2.0	3.1	86.9	0.1	88.6	0.0	89.8	0.1	99.4	0.0	10.1	0.8	9.0	0.8	88.8	0.8	81.0	0.4
2.0	6.3	84.0	0.1	91.0	0.0	77.4	0.1	92.8	0.0	7.1	1.0	8.4	1.0	94.1	1.3	85.9	0.5
2.0	12.5	75.4	0.2	88.4	0.0	74.2	0.2	85.1	0.0	9.5	1.5	5.8	1.5	64.6	2.3	59.0	0.8
2.0	25	63.6	0.3	64.0	0.0	52.3	0.3	61.7	0.0	22.0	2.5	0	2.5	44.1	4.3	40.3	1.3
